# Uneven acute non-alcoholic fatty change of the liver after percutaneous transhepatic portal vein embolization in a patient with hilar cholangiocarcinoma – a case report

**DOI:** 10.1186/s12876-017-0715-5

**Published:** 2017-12-06

**Authors:** Chun-Yi Tsai, Motoi Nojiri, Yukihiro Yokoyama, Tomoki Ebata, Takashi Mizuno, Masato Nagino

**Affiliations:** Division of Surgical Oncology, Department of Surgery, Nagoya University Graduate School of Medicine, 65 Tsurumai-cho, Showa-ku, Nagoya, 466-8550 Japan

**Keywords:** Non-alcoholic fatty liver disease, Portal vein embolization, Hilar cholangiocarcinoma, Biliary tract obstruction, Case report

## Abstract

**Background:**

Portal vein embolization is essential for patients with biliary cancer who undergo extended hepatectomy to induce hypertrophy of the future remnant liver. Over 830 patients have undergone the portal vein embolization at our institution since 1990. Non-alcoholic fatty liver disease is an entity of hepatic disease characterized by fat deposition in hepatocytes. It has a higher prevalence among persons with morbid obesity, type 2 diabetes, and hyperlipidemia. Neither the mechanism of hepatic hypertrophy after portal vein embolization nor the pathophysiology of non-alcoholic fatty liver disease has been fully elucidated. Some researchers integrated the evident insults leading to progression of fatty liver disease into the multiple-hit hypothesis. Among these recognized insults, the change of hemodynamic status of the liver was never mentioned.

**Case presentation:**

We present the case of a woman with perihilar cholangiocarcinoma who received endoscopic biliary drainage and presented to our institute for surgical consultation. A left trisectionectomy with caudate lobectomy and extrahepatic bile duct resection was indicated for curative treatment. To safely undergo left trisectionectomy, she underwent selective portal vein embolization of the liver, in which uneven acute fatty change subsequently developed. The undrained left medial sector of the liver with dilated biliary tracts was spared the fatty change. The patient underwent planned surgery without any major complications 6 weeks after the event and has since resumed a normal life. The discrepancies in fatty deposition in the different sectors of the liver were confirmed by pathologic interpretations.

**Conclusion:**

This is the first report of acute fatty change of the liver after portal vein embolization. The sparing of the undrained medial sector is unique and extraordinary. The images and pathologic interpretations presented in this report may inspire further research on how the change of hepatic total inflow after portal vein embolization can be one of the insults leading to non-alcoholic fatty liver disease/ change.

## Background

Non-alcoholic fatty liver disease (NAFLD) is a specific entity of disease representing fat accumulation in hepatocytes that ranges from simple steatosis to necroinflammatory steatohepatitis [1, 2]. By definition, the diagnosis must be made in patients who have no evidence of excessive alcohol consumption. It is becoming one of the most common hepatic diseases in Western countries, with higher prevalence among persons with morbid obesity or type 2 diabetes [3, 4]. The pathogenesis of NAFLD is not fully elucidated, although several theories have been proposed. Regarding inflammatory steatohepatitis, or non-alcoholic steatohepatitis (NASH), the “two-hit hypothesis” postulates that an additional oxidative injury causes hepatocellular inflammation and fibrosis in patients with existing steatosis [5]. Many potential oxidative insults have been proposed as inducers of this process. Following the accumulation of studies, the more rational “multiple-hit hypothesis” which was composed of several parallel factors contributing to progression of fatty liver change has substituted the relatively simplified “two-hit hypothesis” [6]. Regard the two hypotheses, portal flow discrepancies have not been suggested as an underlying cause.

Patients with hilar cholangiocarcinoma usually present with obstructive jaundice at the time of diagnosis and require extended hepatectomy: hence, two procedures are required before the curative operation. The first is biliary drainage to decrease serum total bilirubin levels, and the second is portal vein embolization (PVE), which is necessary for the induction of hypertrophy of the future remnant liver [7, 8]. We have aggressively treated patients with biliary tract cancer and have used PVE without major complications in over 830 patients since 1990 [9, 10]. Although the hemodynamic changes, especially the changes of portal flow after PVE were enormous, none of these patients developed acute fatty change of the liver after PVE according to our database and literature review. The following case represents an even more extraordinary picture of uneven acute fatty change of the liver after PVE, in which the left medial sector of the liver with biliary tract obstruction was spared the changes. The changes between the affected and the unaffected segments are illustrated via images and confirmed by pathologic interpretations.

## Case presentation

**Fig. 1:**
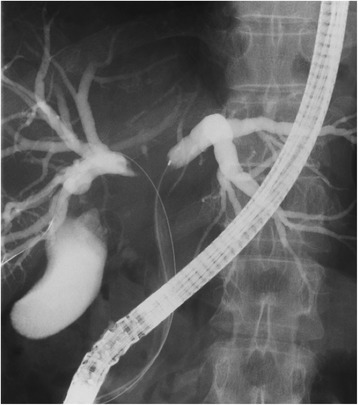
Cholangiographic finding. Cholangiography after ERCP and ENBD showed that the bile ducts of segment IV of the liver were not opacified (drained)

A 55-year-old women who was neither a smoker or heavy drinker fulfilling the criteria for alcoholism was admitted to a regional hospital due to jaundice for 2 weeks. She had underlying hyperlipidemia treated only with diet control. Her body-mass index (BMI) was 26.1 kg/m2. Laboratory tests revealed elevated liver enzymes and hyperbilirubinemia. Computed tomography (CT) demonstrated hilar obstruction with separate bile duct dilatation in the bilateral lobes of the liver. Endoscopic retrograde cholangiopancreatography (ERCP) showed an infiltrating lesion at the bile duct bifurcation, and the diagnosis of hilar cholangiocarcinoma was made after simultaneous endoscopic biopsy. Endoscopic nasobiliary drainage (ENBD) and endoscopic biliary stenting (EBS) were also performed during the same procedure (Fig. 1). She was referred to our hospital for possible surgery. Left trisectionectomy plus caudate lobectomy with extrahepatic bile duct resection was deemed necessary to achieve R0 resection. The future liver remnant (FLR) volume (volume of the right posterior sector) was 423 ml (29.3%) by CT volumetry, and PVE was indicated to induce hypertrophy of the FLR.

**Fig. 2:**
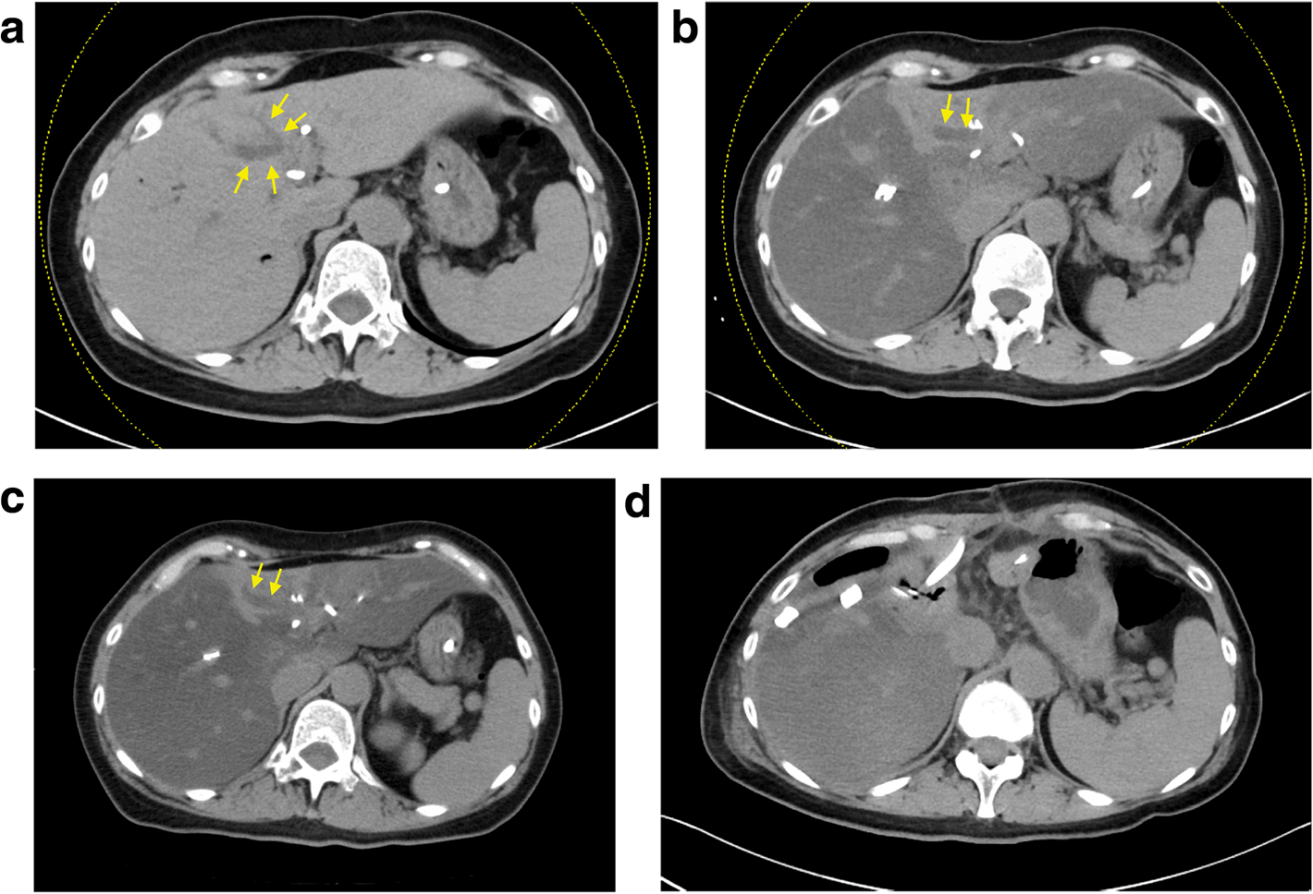
CT of the liver at different stage. **a** CT of the liver before PVE. Yellow arrows, undrained (dilated) bile ducts (**b**) CT after PVE demonstrating acute fatty change of the liver and sparing of segment IV. Yellow arrows, undrained (dilated) bile ducts (**c**) CT 6 weeks after PVE (**d**) CT of the liver remnant 1 week after trisectionectomy

The left and right anterior portal branches were embolized with fibrin and steel coils [9]. There were no complications after the procedure, and routine laboratory testing yielded results similar to those pre-PVE. Routine sonography of the liver was carried out on 5th day after PVE, which confirmed good portal flow in the right posterior sector and thrombosis of the embolized portal branches. The patient was readmitted 3 weeks after PVE for volumetric evaluation of the liver and an indocyanine green clearance (ICG) test. The volume of the right posterior sector had increased to 772 ml (41.3%) and the ICG-K was 0.194. Unexpectedly, a repeat CT showed diffuse fatty change of the liver parenchyma, except for the left medial sector where the bile ducts were incompletely drained (Fig. 2b). Serum laboratory parameters remained unchanged. Surgery was postponed for 3 weeks. The repeat CT still demonstrated uneven fat deposition in the liver (Fig. 2c).

**Fig. 3:**
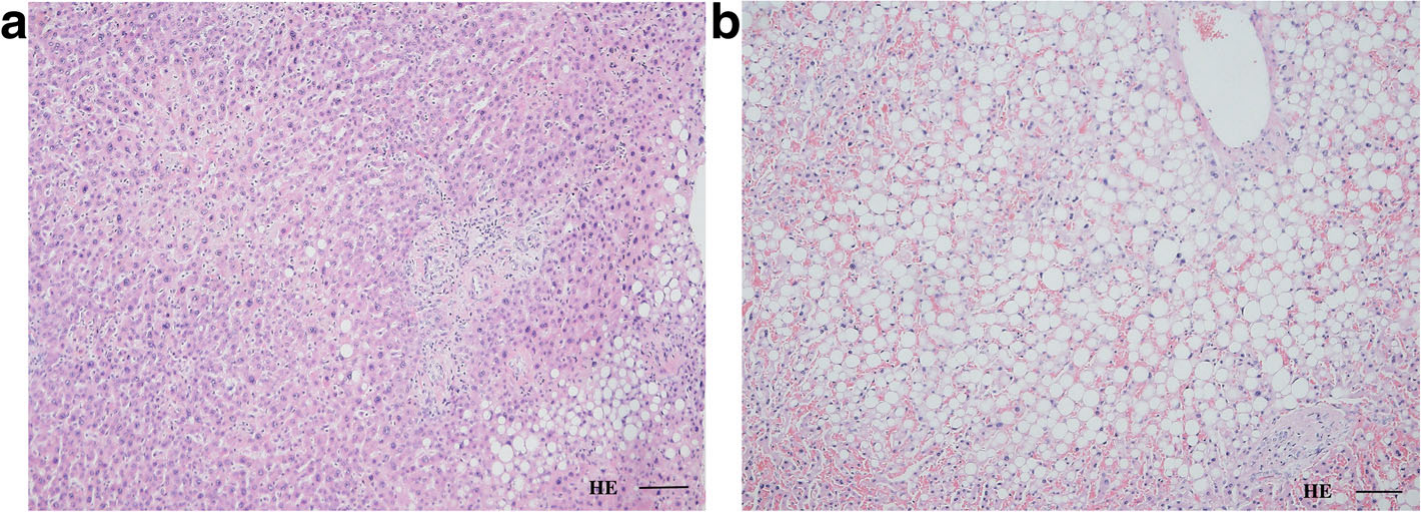
Histologic aspect of different sectors of the liver. Microscopic pictures (100×, H&E stain, scale bar =100 μm) of fatty change in the medial sector (**a**) and lateral sector (**b**). Fat deposition is remarkable in the lateral sector

Due to concern regarding cholangiocarcinoma progression, surgery was performed according to preoperative planning. Gross inspection on laparotomy revealed fatty change of the liver. The pathologic interpretation of the specimen showed different proportions of macrovesicular and microvesicular fat deposition, whereas the left medial sector showed only minimal changes (Fig. 3). The patient did not suffer hepatic dysfunction after surgery. A biliary fistula developed at the transection surface. She was discharged after resolution of the biliary fistula on postoperative day 62 and has since resumed a normal life. The course of treatment was summarized in the Table 1.

**Table 1:** Timeline

July 2015	Jaundice for 2 weeks
August 2015	MDCT, ERCP and biopsy confirmed hilar cholangiocarcinoma
	EBS and ENBD for biliary drainage
August 2015	PVE for left trisectionectomy
September 2015	Unevenly acute fatty change of the liver on MDCT
October 2015	Stationary of fatty change of the liver
	Operation: left trisectionectomy and caudate lobectomy with extrahepatic bile duct resection
	Surgical complication: biliary fistula
November 2015	Discharged from hospital
February 2016	Follow up without recurrence and sequela

*MDCT* multidetector computed tomography, *ERCP* endoscopic retrograde cholangiopancreaticography, *EBS* endoscopic biliary stent, *ENBD* endoscopic nasobiliary drainage, *PVE* portal vein embolization

## Discussion

There were two unusual observations in this case. First, to our knowledge, this is the first case of acute fatty change of the liver after PVE. Second, only the left medial sector of the liver, in which the biliary tracts were incompletely drained, was spared the fatty change, as demonstrated by both imaging and pathologic interpretation of the specimens. Although the mechanisms underlying these observations are unknown, they may have provided a connection between the change of hepatic inflow and the mechanism leading to progression of NAFLD.

NAFLD represents a broad spectrum of disease due to fat deposition in the liver, ranging from asymptomatic hepatic steatosis to necroinflammatory steatohepatitis leading to irreversible cirrhosis [11]. The manifestation of triglyceride accumulation may be due to excessive importation from the blood stream or diminished exportation and oxidation [12]. Steatosis could be detected on images; however, the diagnosis requires the specific histopathologic features demonstrated by biopsy of the liver. To delineate the mechanism of progression of NAFLD to NASH, the “multiple-hit hypothesis” that multiple parallel insults attack on genetic predisposing individuals was proposed to substitute the outdated “two-hit hypothesis” [13]. Regard the patient presented with extraordinary acute fatty deposition of the liver after PVE, which was confirmed by pathologic interpretations, we attempted to integrate the hemodynamic changes in her liver after PVE as one of the possible insults.

Based on a review of her disease course, PVE was the only major event before the uneven fatty changes of the liver. Since 1990, we have performed over 830 PVEs for patients with biliary tract cancer. This is the first patient to develop acute fatty change of the liver after PVE, either from our series or from the literature. PVE induces hemodynamic changes in both arterial and portal blood flow in both embolized and non-embolized hepatic tissue [14]. The volume of her right posterior sector increased from 423 ml (29.4%) to 772 ml (41.3%) after PVE. The volume of the entire liver increased from 1441 ml to 1869 ml after PVE. The effect of PVE was remarkable in this patient.

**Fig. 4:**
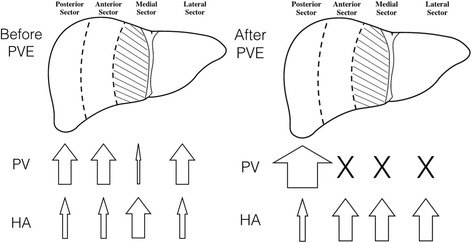
Inflow of the liver before and after PVE. Illustration of the changes in arterial and the portal flow in response to PVE in the patient. PVE, portal vein embolization; PV, portal venous flow; HA, hepatic arterial flow

Alterations in portal and arterial flow secondary to acute biliary tract obstruction have been observed in dogs [15]. The phenomenon is discussed mostly in patients with obstructive jaundice who receive PVE because of its negative impact on FLR hypertrophy [14]. Based on this patient’s CT results and the pathologic interpretations of the specimens, it was confirmed that the incompletely drained medial sector was spared or minimally affected by the acute fatty change after PVE. It was speculated that portal venous flow was substantially decreased because of the dilated bile duct in the Glisson’s sheath before PVE. Therefore, total blood flow in the left medial sector did not change much after PVE, which is in contrast to the dramatic blood flow changes experienced by the other sectors. In the non-embolized right posterior sector (the FLR), portal venous flow increased to accommodate blood flow from the remainder of the gastrointestinal tract after PVE. In the embolized sectors, portal venous flow was shut down, and hepatic arterial flow was increased. Hepatic arterial flow accounts for 25% of total hepatic inflow in humans and increases correspondingly to maintain constant hepatic inflow when portal flow is altered, which is known as the hepatic arterial buffer response [16]. Figure 4 illustrates the indicative, non-quantified changes in both hepatic arterial and portal flow before and after PVE among different sectors of the liver. In summary, the left medial sector experienced the least change in total inflow stemming from dilated biliary trees (unrelieved obstruction).

## Conclusion

In conclusion, the uneven fatty change of the liver in this patient was extraordinary and has never been reported. We surmised that differences in hepatic inflow led to uneven sparing with respect to fatty change of the liver. Although the mechanisms underlying NAFLD and hepatic hypertrophy after PVE involve more than mere hemodynamic changes [5, 14], this specific presentation may give a clue for further research on the hemodynamic change of the hepatic inflow as a potential insult leading to progression of NAFLD.

## Abbreviations

CT: computed tomography
EBS: endoscopic biliary stenting
ENBD: endoscopic nasobiliary drainage
ERCP: endoscopic retrograde cholangiopancreatography
FLR: future liver remnant
ICG: indocyanine green
NAFLD: non-alcoholic fatty liver disease
NASH: non-alcoholic steatohepatitis
PVE: portal vein embolization
